# Book Reviews

**Published:** 1873-12-01

**Authors:** 


### Book Reviews.

Lectures on Diseases and Injuries of the Ear. Delivered at St. George's Hospital, by W. B. Dalby, F.R.C.S. With 21 illustrations. Philadelphia: Lindsay & Blakiston.

This is a small octavo of 224 pages. In the eleven lectures of which it is made up, all the topics appertaining to the subject of diseases and injury of the ear seem to be considered, in a somewhat brief but decidedly clear and practical manner. An abstract of most of these lectures appeared in the *London Lancet* during the year 1872. With a few additions and alterations, the author has gathered them together in the present form, and rendered them sufficiently complete, so that the little volume may serve as a concise, practical textbook for the student or practitioner.

Laceration of the Female Perineum and Vesico-Vaginal Fistula; their History and Treatment. By D. Hayes Agnew, M.D. With numerous illustrations. 8vo. 137 pp. Philadelphia: Lindsay & Blakiston. 1873.

The subjects here presented appeared several years ago—the first in the *Pennsylvania Hospital Reports*, and the second in the pages of the *Medical and Surgical Reporter*, Philadelphia.

The interest and value of the papers, as originally published, causing frequent inquiry to be made for them, the author has been induced to gather them together in the present form. The pages are numerously and finely illustrated, showing the various instruments used in the operations, and their modes of application, etc.

On the Treatment of Diseases of the Skin; with an Analysis of Eleven Thousand Consecutive Cases. By Dr. McCall Anderson. 8vo. 80 pp. Philadelphia: Henry C. Lea. (For sale by Jansen, McClurg & Co., Chicago.)

The author has engrafted a good many new ideas, and a large amount of valuable information regarding the treatment of skin diseases, into this little work.

